# Molecular Characterization of the Cytidine Monophosphate-N-Acetylneuraminic Acid Hydroxylase (*CMAH*) Gene Associated with the Feline AB Blood Group System

**DOI:** 10.1371/journal.pone.0165000

**Published:** 2016-10-18

**Authors:** Toshinori Omi, Shota Nakazawa, Chihiro Udagawa, Naomi Tada, Kazuhiko Ochiai, Yong Hwa Chong, Yuiko Kato, Hiroko Mitsui, Azusa Gin, Hitomi Oda, Daigo Azakami, Kyoichi Tamura, Toshinori Sako, Takeshi Inagaki, Atsushi Sakamoto, Toshihiko Tsutsui, Makoto Bonkobara, Shuichi Tsuchida, Shigenori Ikemoto

**Affiliations:** 1 Department of Basic Science, School of Veterinary Nursing and Technology, Faculty of Veterinary Science, Nippon Veterinary and Life Science University, Tokyo, Japan; 2 International Institute of Small Animal Medicine (Bio Plus), AHB Inc., Tokyo, Japan; 3 Department of Veterinary Nursing, School of Veterinary Nursing and Technology, Faculty of Veterinary Science, Nippon Veterinary and Life Science University, Tokyo, Japan; 4 Department of Veterinary Clinical Pathology, School of Veterinary Medicine, Faculty of Veterinary Science, Nippon Veterinary and Life Science University, Tokyo, Japan; 5 Department of Legal Medicine, Jichi Medical University, Tochigi, Japan; 6 Laboratory of Comparative Cellular Biology, School of Veterinary Medicine, School of Veterinary Medicine, Nippon Veterinary and Life Science University, Tokyo, Japan; 7 School of Veterinary Medicine, Faculty of Veterinary Science, Nippon Veterinary and Life Science University, Tokyo, Japan; Universidade de Sao Paulo, BRAZIL

## Abstract

Cat’s AB blood group system (blood types A, B, and AB) is of major importance in feline transfusion medicine. Type A and type B antigens are Neu5Gc and Neu5Ac, respectively, and the enzyme CMAH participating in the synthesis of Neu5Gc from Neu5Ac is associated with this cat blood group system. Rare type AB erythrocytes express both Neu5Gc and Neu5Ac. Cat serum contains naturally occurring antibodies against antigens occurring in the other blood types. To understand the molecular genetic basis of this blood group system, we investigated the distribution of AB blood group antigens, *CMAH* gene structure, mutation, diplotypes, and haplotypes of the cat *CMAH* genes. Blood-typing revealed that 734 of the cats analyzed type A (95.1%), 38 cats were type B (4.9%), and none were type AB. A family of three Ragdoll cats including two type AB cats and one type A was also used in this study. *CMAH* sequence analyses showed that the CMAH protein was generated from two mRNA isoforms differing in exon 1. Analyses of the nucleotide sequences of the 16 exons including the coding region of *CMAH* examined in the 34 type B cats and in the family of type AB cats carried the *CMAH* variants, and revealed multiple novel diplotypes comprising several polymorphisms. Haplotype inference, which was focused on non-synonymous SNPs revealed that eight haplotypes carried one to four mutations in *CMAH*, and all cats with type B (n = 34) and AB (n = 2) blood carried two alleles derived from the mutated *CMAH* gene. These results suggested that double haploids selected from multiple recessive alleles in the cat *CMAH* loci were highly associated with the expression of the Neu5Ac on erythrocyte membrane in types B and AB of the feline AB blood group system.

## Introduction

Blood group antigens are hereditary polymorphic molecules expressed on the erythrocyte membrane. These antigens are either sugars or proteins, and the antibodies against blood group antigens are either acquired (e.g., produced after transfusion) or naturally occurring [1,2]. Blood group systems are present in humans and in many animal species (e.g., monkey, horse, pig, cattle, sheep, dog, cat, mouse, rabbit, chicken) [3–5].

AB blood group antigens are the most significant in cats’ transfusion medicine and in neonatal isoerythrolysis (NI) [6–10]. The feline AB blood group system consists of A and B antigens and contains blood group type A (type A), blood group type B (type B), and the rare blood group type AB (type AB). Type A erythrocytes express N-glycolylneuraminic acid (Neu5Gc) and type B erythrocytes express N-acetylneuraminic acid (Neu5Ac) [11,12]. Cat serum contains naturally occurring antibodies against the other erythrocyte antigens, i.e.,Type A cats have antibodies to type B antigen, and cats with type B blood have antibodies to type A antigen. Type AB erythrocytes express both Neu5Gc and Neu5Ac, and none of the naturally occurring antibodies to blood type A or B is present [7]. Three alleles, A > a^ab^ >b were proposed to determine the feline AB blood group system [13].

Until now, differences in the distribution of cat AB blood groups have been detected worldwide through studies conducted in North America [10,14–17], South America [18], Oceania [7,19, 20], Asia [21–25], Middle East [26], and Europe [27–41]. According to a survey conducted for 1,985 cats in Brisbane [7], 73.3% were type A, 26.3% were type B, and 0.4% were type AB. In the United Sates, 89% of the 2,172 cats examined were type A and 11% were type B [14]. Among cat breeds, Ginger cats had the lowest frequency of type B blood, and this type was variably high in Abyssinian, Birman, British Shorthair, Devon Rex, Himalayan, Persian, Scottish Fold, and Somali breeds [14]. Ragdoll cats surveyed in Italy presented a high frequency (18%) of blood type AB [39].

The enzyme cytidine monophosphate-N-acetylneuraminic acid hydroxylase is encoded by the *CMAH* gene, which synthesizes Neu5Ac to Neu5Gc [42,43]. Humans only have Neu5Ac because their *CMAH* gene is not functional. This is due to a 92 bp deletion in exon 6 of this gene, a mutation that is estimated to have occurred about two to three millions years ago, before the emergence of the genus *Homo*, Thus, *CMAH* provides information on human evolution [44–47]. In addition, human Neu5Ac is regarded as a receptor for human influenza viruses [48]. Recently, Ng et al. found that Neu5Gc is not synthesized in ferrets, which only express Neu5Ac, pointing these mammals as a unique suitable model for studying the human-adapted influenza A virus [49].

Intact and mutated types of the *CMAH* gene are distributed in cat populations, and associated with the feline AB blood group system. The intact allele (A allele) synthesizes from Neu5Ac to Neu5Gc, and is associated with the type A cats. Because the A allele is dominant relatively to the mutated allele (known as b allele), type A cats can be homozygous, carrying only the A allele, or heterozygous, carrying A and b, while type B cats are homozygous for the b allele [13]. The b allele is a mutated *CMAH* gene with two upstream single nucleotide polymorphisms (SNPs) (-371C>T and -217G>A), a 18 bp deletion (Del)/insertion (Ins) in the 5’ untranslated region (UTR), and three non-synonymous SNPs, including an exon 2 c. 142G>A (Val48Met), an exon 2 c. 268T>A (Tyr90Asn), and an exon 13 c. 1603G>A (Asp535Asn). The variants associated with type AB were not identified in a first report on cat *CMAH* mutations. The position of these SNPs identified in coding regions is numbered up to three bp relative to the original *CMAH* gene, as revealed in exon 1a of the *CMAH* 1a mRNA isoform detected in our study. In a previous experiment, we identified a type B cat not presenting the expected mutations in the *CMAH* gene, suggesting *CMAH* has several variant alleles in cat populations [50].

The present study describes the molecular basis of cat *CMAH* gene variants gene associated with the cat AB blood group system, using serologic assays and sequence analysis. A relationship between *CMAH* variants and the cat blood types B and AB is proposed.

## Materials and Methods

### Ethics Statement

Blood samples from random cat populations were provided by the Department of Veterinary Clinical Pathology, Nippon Veterinary and Life Science University (NVLU), and collected at the Veterinary Medical Teaching Hospital at NVLU, with the written consent of cat owners. Blood samples of a family of Ragdoll cats were provided by the International Institute of Small Animal Medicine (Bio Plus, Japan). Sample collection was only handled by licensed veterinarians. All animal experiments were approved by The Experimental Animal Ethics Committee at NVLU.

### Samples and blood typing

The 776 cats sampled for blood consisted of 773 random cats plus a family of three cats, which included type AB cats, as determined by blood typing. Thirty-eight of these samples were used in cat’s *CMAH* gene expression analysis (S1 Table).

Blood types were determined using RapidVet^®^-H Feline Blood Type Cards (Kyoritsu Seiyaku Corporation, Tokyo, Japan). Briefly, one drop RBC (approximately 50 μl) was placed in the two wells marked with “A”, containing the A (Neu5Gc) antibody, and B, containing lectin (Wheat germ agglutinin) that recognized the B (Neu5Ac) antigen on the feline blood typing card. Blood type A was positive (agglutination) in the well marked “A” and negative (no agglutination) in the well marked “B”. Blood type B was negative in the well marked “A” and positive in the well marked “B”. Blood type AB was positive both wells. In addition, antigens and antibodies were tested by the tube technique. The strength of antigen expression was investigated by double diluting the antibody, eluted from the RapidVet^®^-H Feline blood typing card, using the buffer provided with the kit, and a 3% red blood cells (RBCs) solution. The strength of the natural antibody contained in the serum was investigated by double diluting the serum with physiological saline and 3% RBCs solutions. Each antibody or serum mix were then added to 20 μl 3% RBCs solution in 12 x 75 mm tubes, at room temperature, and centrifuged at 3,000 rpm for 15 seconds. Agglutination was considered positive if RBCs remained agglutinated after tubes were gently shaken.

### *CMAH* cDNA cloning

Cat’s *CMAH* cDNA was synthesized from the RNA isolated from whole blood samples by reverse-transcription PCR (RT-PCR), using the Reverse Transcription System (Promega Corporation, Madison, WI, USA) in 25 μl total reaction volumes, and following the manufacturer’s instructions. Overlapping fragments including open reading frames (ORFs) were amplified using FastStart *Taq* DNA polymerase (Roche Diagnostics, Mannheim, Germany) and the set of primers and RT-PCR conditions shown in S2 Table. Sequencing was performed directly on purified RT-PCR products (Roche) or on purified plasmid DNA, which was obtained by inserting cDNA fragments into a TOPO^®^ TA plasmid vector (Invitrogen, Carlsbad, CA, USA).

### PCR amplification of *CMAH* from genomic DNA

Genomic DNA was extracted from 38 blood samples using the Puregene kit (Qiagen, Valencia, CA, USA), according to the manufacturer's instructions. Sixteen exons (Exons 1a and 1b, and Exons 2 to 15) of the *CMAH* gene, containing a 5’ UTR and a coding region, were amplified from the genomic DNA of one type A cat (used as control), 34 type B cats, and from a family of three Ragdoll cats including type AB cats. Information on these blood samples is shown in S1 Table. PCR primers were designed using a cat *CMAH* sequence (NC_018727.1), *CMAH* alleles of type A cats obtained from an mRNA sequence (EF127684.1), and from a *CMAH* variant in type B cats with a previously described mutation [13].

Using the FastStart *Taq* DNA polymerase (Roche) and 25 μl total reaction volumes, prepared according to manufacturer's instructions (Roche), PCRs were performed on a TaKaRa PCR Thermal Cycler Dice TP600 (TaKaRa Bio Inc., Shiga, Japan), following the conditions and using the primers shown in S2 Table. Amplification, was confirmed on 2% agarose gels stained with ethidium bromide (Nippon Gene Co., Ltd., Toyama, Japan).

### Sequencing and Mutation detection

Sequencing was performed directly on RT-PCR or PCR products, purified using the High Pure PCR Product Purification Kit (Roche), or on purified DNA plasmids obtained from TOPO^®^ TA vectors (Invitrogen). Using the BigDye Terminator kit v3.1, sequencing was performed on an ABI 310 or a 3730 Genetic Analyzer (all from Applied Biosystems, Foster City, CA, USA). BigDye Xterminator Purification kits were used according to the manufacturer’s instructions (Applied Biosystems) to purify dye-labeled fragments.

We identified DNA polymorphisms by comparing each sequence obtained with the sequences used to design primers (*i*.*e*., NC_018727.2; cat chromosome B alleles A—EF127684.1 and B—EF127685.1), and using the basic local alignment search tool (BLAST) at the National Center for Biotechnology Information (NCBI) website. The program GENETYX v11 (GENETYX Corporation, Tokyo, Japan) was used to analyze sequences. The position of SNPs located in coding regions was numbered from the A of the initiator methionine (ATG) codon, as revealed in exon 1a of the *CMAH* 1a mRNA isoform described in the present study, located 3 bp upstream of the original position reported [13].

### Haplotype analysis

We performed the estimation of haplotypes from genotype data using the software SNPAlyze v8 (DYNACOM Co., Ltd., Chiba, Japan). Relationships among haplotypes were evaluated in haplotype networks constructed using TCS v1.21 [51]. The sequence data used as input for haplotype networks were reconstructed from exon sequences considering amino acid substitutions.

## Results

### Blood typing in the cats AB blood group system

The 773 cats blood-typed according to the feline AB blood type system, comprised 291 pure-bred cats belonging to 26 breeds (one to 86 cats in each breed), and 482 cats belonging to an unidentified breed or hybrid. Blood-typing revealed that 735 cats were type A (95.1%), 38 cats were type B (4.9%), and none were type AB (Table 1). S3 Table shows the detailed results of blood-typing.

**Table 1:** Blood typing of feline AB blood group antigens in a random population.

Animals	N	Type A	Frequency	Type B	Frequency	Type AB	Frequency
		Neu5Gc	%	Neu5Ac	%	Neu5Gc Neu5Ac	%
Pure-bred	291	270	92.8	21	7.2	0	0
Unidentified breed or Hybrid	482	465	96.5	17	3.5	0	0
Total	773	735	95.1	38	4.9	0	0

As type AB cats were not found in the randomly selected cat populations, we tried to find AB blood type cats among one of the pure breeds where this type of blood has been found, *i*.*e*., Ragdoll cats [39]. The putative type AB cat found (male proband) was tested using RBCs at about two-months-old and seven-months-old, and revealed type AB blood on both instances. The serum of this AB male proband did not agglutinate with type A and type B RBCs, corroborating its characterization as a type AB cat. The blood type of the proband’s parents (Ragdoll cats) was also determined: the mother was type AB and the father was type A (Table 2).

**Table 2:** Serological tests of the Ragdoll cat family with blood type AB.

Animals	Breed	RBCs		Serum		Blood type
		Anti-Neu5Gc (A)	Anti-Neu5Ac (B)	A type RBCs	B type RBCs	
Family						
Proband	Ragdoll	positive	positive	negative	negative	AB
Mother	Ragdoll	positive	positive	negative	negative	AB
Father	Ragdoll	positive	negative	negative	positive	A
Control						
Type A		positive	negative	negative	positive	A
Type B		negative	positive	positive	negative	B

### *CMAH* cDNA cloning

**Fig 1:**
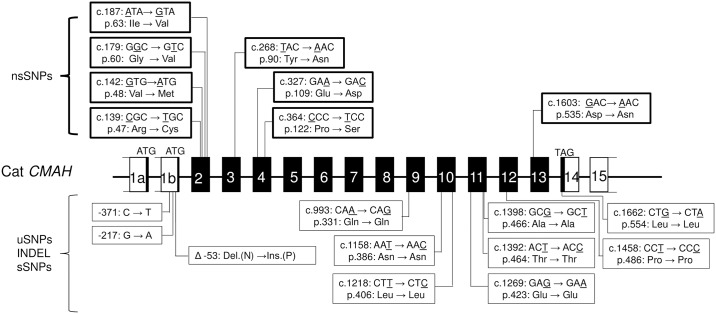
Structure of cat’s *CMAH* gene. (A) Genomic structure of cat’s *CMAH* gene obtained from the comparative analysis of mRNA sequences and whole genome shotgun sequence (Accession No.NC_018727.2, chromosome B). Exon 1a is located upstream of exon 1b. *CMAH* 1a mRNA corresponds to the mRNA sequences deposited in Genbank under Accession No. EF127684.1 from blood type A cat, while *CMAH* 1b mRNA corresponds to first presorted cat *CMAH* mRNA from blood type A cat [13]. □: Exon(UTR), ■: Exon(CDS), ▬: Intron. (B) Nucleotide sequences of the 5’ region of two mRNA isoforms, which have different exons (exon 1a and exon 1b) with start codon ATG and are considered leader exons Both exons were spliced to exon 2 to produce the full length cat *CMAH* mRNA isoforms 1a (*CMAH* 1a) and 1b (*CMAH* 1b).

Because there were two full-length cat *CMAH* cDNA sequences derived from different leading exons in a previously report [13] and only one mRNA sequence retrieved from GenBank (EF127684.1), it was not clear if tthe same individual carried two kinds of cDNA encoding CMAH. Thus, we investigated cDNAs sequences encoding the intact CMAH protein in type A cats. We found two cDNA isoforms in the same individual with type A blood, which derived from different leading exons, namely exon 1a (11 bp) and exon 1b (9 bp). Both exons 1a and 1b spliced exon 2 directly, and generated the full-length *CMAH* mRNA isoforms 1a (*CMAH* 1a) and 1b (*CMAH* 1b). Comparisons of the genomic structure of cat *CMAH* gene obtained from mRNA sequencing with whole genome shotgun sequences (NC_018727.2, cat chromosome B) showed that exon 1a was located upstream of exon 1b (Fig 1). The ORFs of these *CMAH* mRNA isoforms included 15 exons. We considered that the *CMAH* 1a mRNA encoding 578 amino acids corresponded to *CMAH* mRNA EF127684.1, while the *CMAH* 1b mRNA encoding 577 amino acids was the first identified cat *CMAH* mRNA [13] from type A cats. These results indicated that the intact CMAH cat protein generated from two highly homologous mRNA isoforms differed by only one exon. In type B cats, the mRNA variant (EF127685.1) based on *CMAH* 1b was reported by Bighignoli et. al. [13], and mRNA variants based on *CMAH* 1a from type B cats found in the present study were submitted to NCBI GenBank as LC051631.1, LC051632.1, and LC051633.1 in April 2015. These results suggested that the region of critical mutation associated with the variant types (type B and type AB) in cat AB blood group was in common coding region of both *CMAH* 1a and *CMAH* 1b mRNAs.

### Mutations in the cat *CMAH* gene

**Fig 2:**
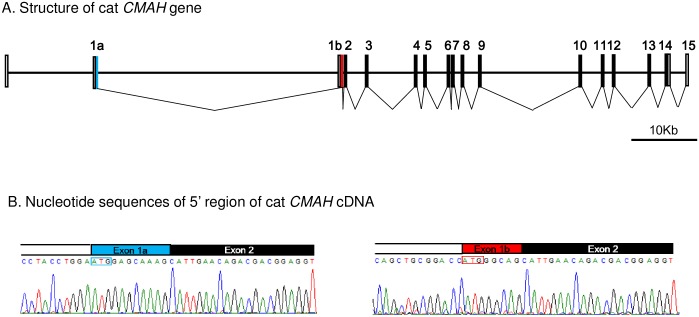
Schematic representation of the DNA polymorphisms detected in the *CMAH* gene in cat. □: Exon(UTR) ■: Exon(CDS) ▬: Intron: _ SNP. The position of identified DNA polymorphism was numbered from nucleotide A of the initiator methionine ATG codon revealed in exon 1a.

To identify *CMAH* variants, 16 exons (exons 1a and 1b, and exons 2 to 15) of the *CMAH* gene, containing a 5’ UTR, a coding region, and a 3’ UTR, were amplified from the genomic DNA of 38 cats: one type A cat (used as control), 34 type B cats, and a family of three Ragdoll cats including two type AB cats and one type A cat (S1 Table). The position of the SNPs identified in coding regions was numbered from nucleotide A within the initiator methionine (ATG) codon, located in exon 1a of the *CMAH* 1a mRNA isoform, as presented in our study. Sequence analysis revealed total of 19 mutations in exons 1b, 2, 3, 4, 9, 10, 11, 12, 13, and 14 but not in exons 1a, 5, 6, 7, 8, and 15 of the cat *CMAH* gene (Fig 2). These mutations were: two untranslated SNPs (uSNPs), -371C>G, -217G>A; one insertion/deletion polymorphism (InDel), Δ-53 N (18b insertion)>P (18bp deletion) in exon 1b; eight non-synonymous SNPs (nsSNPs), c.139C>T (Arg47Cys), c.142G>A (Val48Met), c.179G>T (Gly60Val), and c.187A>G (IIe63Val) in exon 2, c.268T>A (Tyr90Asn) in exon 3, c.327A>C (Glu109Asp), and c.364C>T (Pro122Ser) in exon 4, and c.1603G>A (Asp535Asn) in exon 13; and eight synonymous SNP (sSNPs), c.933A>G (Gln331Gln) in exon 9, c.1158T>C (Asn386Asn) and c.1218T>C (Leu406Leu) in exon 10, c.1269G>A (Glu423Glu), c.1392T>C (Thr464Thr), and c.1398G>T (Ala466Ala) in exon 11, c.1458T>C (Pro486Pro) in exon 12, and c.1662G>A (Leu554Leu) in exon 14. Among the nsSNPs, c.142G>A, c.268T>A, c.327A>C, c.364A>C, and c.1603G>A were previously identified by Bighignoli et. al. [13], Gandolfi et. al. [52], and in the cDNA analysis developed in this study (LC051632.1, and LC051633.1). The SNP c.139G>A was previously identified by Tasker et. al. [53] from genomic DNA. Two uSNPs and one InDel (-371C>G, -217G>A, Δ-53 N>P) and six nsSNPs (c.933A>G, c.1158T>C, c.1269G>A, c.1392T>C, c.1398G>T, and c.1458T>C) have been detected in the first report of the cat *CMAH* mutations (EF127685.1) [13]. Two nsSNPs (the c.179G>T and c.187A>G) and two sSNPs (the c.1218T>C, and c.1662G>A) were newly detected in this study from genomic DNA sequences in this study.

### Diplotypes of the cat *CMAH* gene

Thirteen diplotypes derived from 19 mutations (two uSNPs, one InDel, eight nsSNPs, and eight sSNPs) detected in this study (Table 3). Diplotypes 1 and 2 corresponded to the previous *CMAH* alleles, which were reported from the mRNA type A cats sequence EF127684.1, and to the first causative *CMAH* variant in type B cats [13]. The remaining 11 diplotypes (Diplotypes 3 to 13) were novel to cat’s *CMAH* loci. Diplotypes had four to 14 mutations in different nucleotide positions and there was was no common mutation among the 13 diplotypes.

**Table 3:** Identification of Diplotypes and their mutations in cat’s *CMAH* gene.

			E1b(5'UTR)			E2				E3	E4		E9	E10		E11			E12	E13	E14
Diplotype	Blood type	N	-371	-217	Δ-53	c.139	c.142	c.179	c.187	c.268	c. 327	c. 364	c.993	c.1158	c.1218	c.1269	c.1392	c.1398	c.1458	c.1603	c.1662
			C>T	G>A	N>P	C>T	G>A	G>T	A>G	T>A	A>C	C>T	A>G	T>C	T>C	G>A	C>T	G>T	T>C	G>A	G>A
						R47C	V48M	G60V	I63V	Y90N	E109D	P122S	Q331Q	N386N	L406L	E423E	T464T	A466A	P486P	D535N	L554 L
Dip 1*	**A**	**1**	**CC**	**GG**	**NN**	**CC**	**GG**	**GG**	**AA**	**TT**	**AA**	**CC**	**AA**	**TT**	**TT**	**GG**	**CC**	**GG**	**TT**	**GG**	**GG**
**Dip 2**	**B**	**19**	**TT**	**AA**	**PP**	**CC**	**AA**	**GG**	**AA**	**AA**	**CC**	**CC**	**AA**	**CC**	**TT**	**AA**	**TT**	**GG**	**TT**	**AA**	**GG**
**Dip 3**	**B**	**5**	**TT**	**AA**	**PP**	**CT**	**GA**	**GT**	**AA**	**AA**	**CC**	**CC**	**AA**	**CC**	**TC**	**GA**	**CT**	**GG**	**TC**	**GA**	**GG**
**Dip 4**	**B**	**4**	**CT**	**GA**	**NP**	**CC**	**GA**	**GG**	**AA**	**TA**	**AC**	**CT**	**AA**	**CC**	**TT**	**AA**	**TT**	**GT**	**TT**	**GA**	**GG**
**Dip 5**	**B**	**1**	**CT**	**GA**	**NP**	**CC**	**GA**	**GG**	**AA**	**TA**	**CC**	**CC**	**AA**	**CC**	**TT**	**AA**	**TT**	**GT**	**TT**	**GA**	**GG**
**Dip 6**	**B**	**1**	**CT**	**GA**	**NP**	**CC**	**GA**	**GG**	**AA**	**TA**	**CC**	**CC**	**AG**	**CC**	**TT**	**GA**	**CT**	**GG**	**TT**	**GA**	**GG**
**Dip 7**	**B**	**1**	**CC**	**GG**	**NN**	**CC**	**GA**	**GG**	**AA**	**TA**	**AA**	**CT**	**AA**	**CC**	**TT**	**AA**	**TT**	**GT**	**TT**	**GA**	**GG**
**Dip 8**	**B**	**1**	**CC**	**GG**	**NN**	**CT**	**GG**	**GT**	**AA**	**TT**	**CC**	**CC**	**AA**	**TC**	**TC**	**GG**	**CC**	**GG**	**CC**	**GG**	**GG**
**Dip 9**	**B**	**1**	**CC**	**GG**	**NN**	**CT**	**GG**	**GT**	**AA**	**TT**	**AC**	**CT**	**AA**	**CC**	**TC**	**GA**	**CT**	**GT**	**TC**	**GG**	**GG**
**Dip 10**	**B**	**1**	**CC**	**GG**	**NN**	**CC**	**GG**	**GG**	**AG**	**TT**	**AA**	**CT**	**AA**	**TC**	**TT**	**GG**	**CC**	**GG**	**TT**	**GG**	**AA**
Dip 11**	**AB**	1*	**CC**	**GG**	**NN**	**CC**	**GG**	**GG**	**AG**	**TT**	**CC**	**CC**	**GG**	**CC**	**TT**	**GG**	**CT**	**GG**	**TC**	**GG**	**GG**
Dip 12**	**AB**	1*	**CT**	**GA**	**NP**	**CC**	**GA**	**GG**	**AG**	**TA**	**CC**	**CC**	**AG**	**CC**	**TT**	**GA**	**CT**	**GG**	**TT**	**GA**	**GG**
Dip 13**	**A**	1*	**CC**	**GG**	**NN**	**CC**	**GG**	**GG**	**AA**	**TT**	**AC**	**CC**	**GG**	**TC**	**TT**	**GG**	**CT**	**GG**	**CC**	**GG**	**GG**

Bignoni et al. reported genotypes at -371, -271, Δ-53, c.142 (original c.139), c.268 (original c.265), and c.1603 (original c.1600) [13].

*Diplotype 1 is the intact cat *CMAH* gene.

**Ragdoll family (Diplotype 11 in son, Diplotype 12 in mother, Diplotype 13 in father); Dip: Diplotype; E: exon.

Among type B cats (Diplotypes 2 to 10), Diplotype 2 was the most frequent (55.9%) and eight novel diplotypes (Diplotypes 3 to 10) were distributed in 41.1% of the samples. These results showed that the *CMAH* variant associated with type B blood was not only the main cause of *CMAH* variation as a first reported *CMAH* variant (Diplotype 2) [13], but also that there were several different mutations in the cat’s *CMAH* loci. In addition, the novel diplotypes (Diplotypes 3 to 10) found in type B cats carried the heterozygous mutations.

We tried to investigate the serological differences between Diplotypes 2 (most common) and 7 in type B cats. Erythrocytes’ B antigen and the anti-A antibodies tittered in the serum of type B showed that Diplotype 2 cat were two- and 64-times higher than in Diplotype 7 cat, respectively. These results suggested that the amount of antigen and antibody differed among diplotypes in type B cats expressing Neu5Ac.

We also identified three novel diplotypes (Diplotypes 11 to 13) in the Ragdoll cats family comprising two type AB and one type A cats (Table 3). Focusing on mutation Δ-53 in 5’ UTR and on the nsSNPs, the genotype of the type AB male proband was genotype NN at Δ-53N>P, AG at c.187A>G, and CC at c.327 A>C (Diplotype 11). The genotype of the proband’s mother, also a type AB cat, was NP at Δ-53 N>P, GA at c.142G>A, AG at c.187A>G, TA at c.268T>A, CC at. c. 327A>C, and GA at c.1603G>A (Diplotype 12). The proband’s father (type A cat) had a variant C allele at c.327 (Diplotype 13).

The genotype CC at. c.327A>C was found in the two type AB cats (proband and proband’s mother) was also detected as the most frequent mutation in type B diplotypes (Diplotypes 2, 3, 5, 6, and 8). Thus, the mutation c.327A>C alone does not determine type AB cats but might be highly associated with the expression of Neu5Ac in these cats. In addition, Gandolfi et al. have recently (2016) reported a novel variant with the mutation c.364C>T in type AB in Ragdoll cats [52], but the novel variants identified in the type AB Ragdoll cats examined in the present study differed from that. Overall, mutation data showed that different genotypes generate different blood type AB cats, similar to that found for type B cats.

### Haplotypes in cat *CMAH* gene

**Fig 3:**
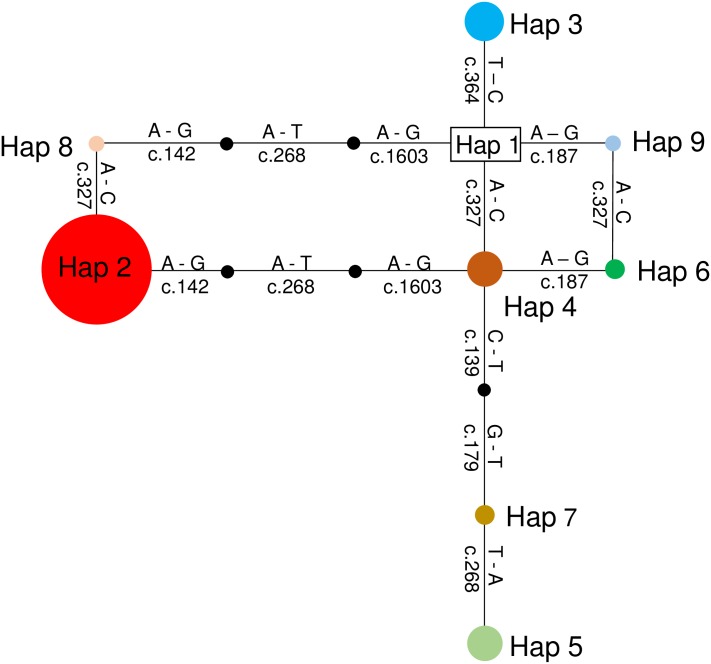
Relationships among cat’ *CMAH* gene haplotypes. The relationships among haplotypes were based on the haplotype networks obtained in TCS software [51]. Each circle is proportional to the haplotype frequency. Each small white circle represents an intermediate non-sampled or non-existent haplotype. Each line within the *CMAH* network represents a mutation.

The nine haplotypes inferred from the cat *CMAH* gene by maximum parsimony corresponded to the eight nsSNPs (c.139C>T, c.142G>A, c.179G>T, c.187A>G, c.268T>A, c.327A>C, c.364 C>T, and c.1603G>A) (Table 4). Haplotypes 1 and 2 corresponded to the intact *CMAH* allele (Type A) and to the first causative mutation leading to *CMAH* variants (Type B), respectively [13]. The remaining seven haplotypes (Haplotypes 3 to 9) were novel to the cat *CMAH* gene. These haplotypes had one to four mutations in coding regions. Haplotype 2 was the most frequent haplotype (72.1%) among type B cats. The schematic representation of the relationships between haplotypes obtaine in TCS is shown in Fig 3.

**Table 4:** Haplotypes inferred by maximum parsimony from eight nsSNPs in cat’s *CMAH* gene.

	E2				E3	E4		E13
Haplotype	c.139	c.142	c.179	c.187	c.268	c.327	c.364	c.1603
	C>T	G>A	G>T	A>G	T>A	A>C	C>T	G>A
	R47C	V48M	G60V	I63V	Y90N	E109D	P122S	D535N
Hap 1*^†^	C	G	G	A	T	A	C	G
Hap 2^†^	C	A	G	A	A	C	C	A
Hap 3^†^	C	G	G	A	T	A	T	G
Hap 4	C	G	G	A	T	C	C	G
Hap 5	T	G	T	A	A	C	C	G
Hap 6	C	G	G	G	T	C	C	G
Hap 7^†^	T	G	T	A	T	C	C	G
Hap 8	C	A	G	A	A	A	C	A
Hap 9	C	G	G	G	T	A	C	G

*Haplotype 1 is the intact cat *CMAH* gene.

^†^Haplotypes 1, 2, 3, and 7 were also detected in cDNA analysis.

Diplotypes 1 to 13 shown in (Table 3) were categorized according to their amino acid substitutions (Table 5) using the software SNPAlyze (DYNACOM Co., Ltd.). Haplotype combinations of 1–1 in Diplotype 1 were considered the intact type (type A). In type B cats, haplotype combinations were categorized 2–2 (Diplotype 2), 2–5 (Diplotype 3), 2–3 (Diplotype 4), 2–4 (Diplotypes 5 and 6), 3–8 (Diplotype 7), 4–7 (Diplotype 8), 3–7 (Diplotype 9), and 3–9 (Diplotype 10). Among the family of Ragdoll cats, haplotype combinations were 4–6 (Diplotype 11 from Type AB), 2–6 (Diplotype 12 from Type AB), and 1–4 (Diplotype 13 from Type A). Cats with blood types B and AB (Diplotypes 2 to 12), which expressed Neu5Ac on erythrocytes, did not carriy the intact *CMAH* gene haplotype (Haplotype 1). Detailed information on individuals’ diplotypes and haplotype are shown in S4 and S5 Tables. These results suggested that double haploids selected from multiple recessive alleles in the cat *CMAH* loci were highly associated with the expression of the Neu5Ac on the erythrocyte membrane in the feline AB blood group system.

**Table 5:** Amino acid substitutions in diplotypes and inferred haplotypes in cat’s *CMAH* gene.

	E2				E3	E4		E13
Haplotype	c.139	c.142	c.179	c.187	c.268	c.327	c.364	c.1603
	C>T	G>A	G>T	A>G	T>A	A>C	C>T	G>A
	R47C	V48M	G60V	I63V	Y90N	E109D	P122S	D535N
Dip 1	RR	VV	GG	II	YY	EE	PP	DD
Hap 1	R	V	G	I	Y	E	P	D
Hap 1	R	V	G	I	Y	E	P	D
Dip 2	RR	MM	GG	II	NN	DD	PP	NN
Hap 2	R	M	G	I	N	D	P	N
Hap 2	R	M	G	I	N	D	P	N
Dip 3	RC	VM	GV	II	NN	DD	PP	DN
Hap 2	R	M	G	I	N	D	P	N
Hap 5	C	V	V	I	N	D	P	D
Dip 4	RR	VM	GG	II	YN	ED	PS	DN
Hap 2	R	M	G	I	N	D	P	N
Hap 3	R	V	G	I	Y	E	S	D
Dip 5,6	RR	VM	GG	II	YN	DD	PP	DN
Hap 2	R	M	G	I	N	D	P	N
Hap 4	R	V	G	I	Y	D	P	D
Dip 7	RR	VM	GG	II	YN	EE	PS	DN
Hap 3	R	V	G	I	Y	E	S	D
Hap 8	R	M	G	I	N	E	P	N
Dip 8	RC	VV	GV	II	YY	DD	PP	DD
Hap 4	R	V	G	I	Y	D	P	D
Hap 7	C	V	V	I	Y	D	P	D
Dip 9	RC	VV	GV	II	YY	ED	PS	DD
Hap 3	R	V	G	I	Y	E	S	D
Hap 7	C	V	V	I	Y	D	P	D
Dip 10	RR	VV	GG	IV	YY	EE	PS	DD
Hap 3	R	V	G	I	Y	E	S	D
Hap 9	R	V	G	V	Y	E	P	D
Dip 11	RR	VV	GG	IV	YY	DD	PP	DD
Hap 4	R	V	G	I	Y	D	P	D
Hap 6	R	V	G	V	Y	D	P	D
Dip 12	RR	VM	GG	IV	YN	DD	PP	DN
Hap 2	R	M	G	I	N	D	P	N
Hap 6	R	V	G	V	Y	D	P	D
Dip 13	RR	VV	GG	II	YY	ED	PP	DD
Hap 1	R	V	G	I	Y	E	P	D
Hap 4	R	V	G	I	Y	D	P	D

## Discussion

The feline AB blood group is the most important blood group system in cat transfusion medicine. This blood group system is linked to the cat CMAH protein [11–13] and Neu5Gc and Neu5Ac are the epitopes of antigens A and B blood types, respectively. In the present study, we identified multiple nsSNPs, diplotypes, and haplotypes in cat *CMAH* variants sequenced for 34 type B cats and for a family of two type AB cats and one type A cat.

The loss of enzyme activity in the CMAH enzyme controlling the expression of Neu5Ac on erythrocytes carrying type B antigens derived from mutations in the cat *CMAH* gene [13]. We here first reported that multiple diplotypes, which are characterized by several mutations, could be associated with Neu5Ac expression (Table 3). Furthermore, haplotype inference analysis using the identified diplotypes revealed that type B and AB cats expressing Neu5Ac on erythrocytes carried homozygote *CMAH* variants or heterozygotes with two different *CMAH* variants (Table 5). Thus, the present study suggested that the expression of Neu5Ac in the sampled B and AB cats occurred by combining double haploids selected from various *CMAH* haplotypes where amino acids have changed. The substituted amino acids in cat *CMAH* gene might suppress CMAH activity synthesized Neu5Gc and express Neu5Ac by altering the conformation of the protein, although different amino acids might influence CMAH’s activity differently.

Neu5Gc on cat erythrocytes has been reported to comprise a total of 98%, 65%, and 35–60% of total sialic acid, according to gas-liquid chromatography or reverse-phase high performance liquid chromatography with fluorescent detection [54, 55]. Thin-layer chromatography immunostaining analysis showed that type AB erythrocytes had less Neu5Ac than type A erythrocytes and less Neu5Gc than type B erythrocytes [11]. Until now, expression level of Neu5Gc in types A or AB erythrocytes agglutinated with Neu5Gc monoclonal antibodies had not been systematically determined. The "quantification" and comparisons of Neu5Gc and Neu5Ac in erythrocytes with the blood types, A, B, and AB erythrocytes, and the mutations in the of cat *CMAH* gene obtained here are therefore essential for further genetic analysis of the feline AB blood groups in future.

**Fig 4:**
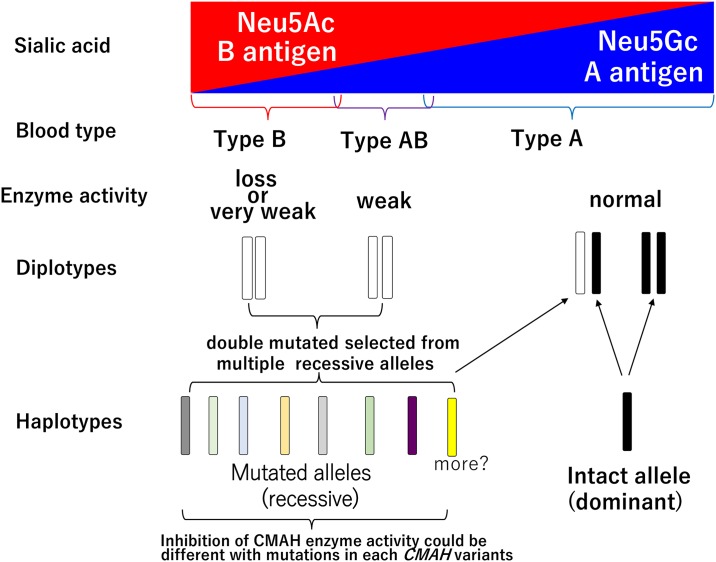
Conceptual scheme of the molecular basis of the feline AB blood group system. □: *CMAH* mutated alleles (recessive), ■: *CMAH* intact allele (dominant), color boxes: haplotypes revealed in types B and AB within cats expressing Neu5Ac. These results suggested that double haploids selected from multiple recessive alleles in cat’s *CMAH* loci were highly associated with the determination of blood types B and AB in the feline AB blood group systems.

Cat’s AB blood group system blood type is generally determined using RBCs card agglutination in relation to antibodies. Recently, Seth et al. [17] compared the card agglutination (CARD), immunochromatographic cartridge (CHROM), gel-based (GEL), and conventional slide (SLIDE) and tube (TUBE) agglutination assays, and found that the sensitivity of the CARD method is 93.2% for A antigen detection and 95.7% for B antigen detection against TUBE method. These data suggested that the discrimination of positive and negative cells by qualitative assessment might be difficult if the expression of antigens is very weak. The present blood types was determined by the CARD method was used in the presented study, and we cloud not exclude the small possibility of phenotypic error when determining blood types. Fig 4 is one of the model to our understanding the molecular basis of the feline AB blood group system, which is based on previous research [7, 11–13, 17, 52, 54–55] and this study in the present. We cannot exclude the possibility that other further mutation might be present in the *CMAH* gene associated with Neu5Ac expression on cat erythrocytes. The exact classification of the blood group by DNA test based on the *CMAH* mutations need to further information for the phenotypes and genotypes characterizing cat’s AB blood type.

Humans do not synthesize Neu5Gc, and only express Neu5Ac, as the human *CMAH* gene lost this function due to the deletion of a 92 bp exon [44–47]. In ferrets, CMAH enzyme activity was lost due to the deletion of nine exons (exons 1 to 9) in the ferret *CMAH* gene [49]. The results of Bighignoli et al. [13] and the present study, showed that the *CMAH* gene carried in type B cats with Neu5Ac expression had no exon deletion, instead showing spontaneous mutations in the protein coding region. These findings suggested that the genetic mechanism inactivating the *CMAH* gene in felines might differ from that in human and ferret. In humans, Neu5Ac is not associated with human blood group system, but is regarded as an important receptor for influenza viruses [48].

Despite the low frequency of blood types B (expressing Neu5Ac) and AB (expressing Neu5Ac + Neu5Gc) in cat populations, the Neu5Ac derived from *CMAH* mutations is still maintained in cat populations. Löfling et al. have recently (2013) reported that canine and feline parvoviruses preferentially recognize Neu5Gc [55]. Thus, considering our findings, mutations in *CMAH* gene revealed in types B and AB may occur due to a selection pressure to maintain Neu5Ac expression against viruses in cat populations [55].

## Conclusions

We identified multiple nsSNPs, diplotypes, and haplotypes in cat *CMAH* variants sequenced for 34 type B cats and for a family of two type AB cats and one type A cat. In our cat samples, the combination of double haploids selected from various recessive alleles with amino acid substitution in cat *CMAH* loci was highly associated with the generation of blood types B and AB expressing the Neu5Ac in the feline AB blood group systems.

## Supporting Information

S1 Table Samples used in the genetic analysis of cat *CMAH*. (PDF)

S2 Table Primer sequences and PCR conditions. (PDF)

S3 Table Distribution of feline AB blood group antigens among the randomly selected cats. (PDF)

S4 Table Frequency of blood type B diplotypes and haplotypes. (PDF)

S5 Table Summary of diplotypes and haplotypes in the Ragdoll cats family with AB and A blood groups. (PDF)
