# Supplementary material for: Molecular Characterization of the Cytidine Monophosphate-N-Acetylneuraminic Acid Hydroxylase (CMAH) Gene Associated with the Feline AB Blood Group System
Source: PLoS One. 2016 Oct 18;11(10):e0165000. doi: 10.1371/journal.pone.0165000 (PMC5068781; doi:10.1371/journal.pone.0165000)
Supplement: S1 Table — (PDF) [file pone.0165000.s001.pdf]

S1 Table. Samples used in the genetic analysis of cat *CMAH*.

Sample	Lab ID	Sex	Breed	Blood type	Analysis
Wild type (Type A cat)					
1	AHB-5	Male	Ragdoll	A	G,C
Type B cats					
2	Gu-019	Male	Tonkinese	B	G,C
3	12742-KA-018	Male	Japanese domestic Cats	B	G,C
4	7231-001	Female	Persian	B	G
5	7909-004	Female	Japanese domestic Cats	B	G
6	8511-006	Female	Scottish Fold	B	G
7	8745-007	Female	Abyssinian	B	G
8	0824-008	Male	Japanese domestic Cats	B	G
9	9724-010	Male	an unidentified breed or hybrid	B	G
10	12534-012	Male	an unidentified breed or hybrid	B	G
11	12657-013	Male	an unidentified breed or hybrid	B	G
12	12896-014	Female	an unidentified breed or hybrid	B	G
13	12958-015	Male	an unidentified breed or hybrid	B	G
14	13259-016	Male	an unidentified breed or hybrid	B	G
15	12962-017	Male	American Shorthair	B	G
16	14348-022	Female	Japanese domestic Cats	B	G
17	15114-024	Female	Japanese domestic Cats	B	G
18	15567-025	Female	an unidentified breed or hybrid	B	G
19	15618-026	Male	an unidentified breed or hybrid	B	G
20	151717-027	Male	an unidentified breed or hybrid	B	G
21	16137-028	Male	an unidentified breed or hybrid	B	G
22	14773-029	Female	an unidentified breed or hybrid	B	G
23	16219-030	Female	an unidentified breed or hybrid	B	G
24	16227-031	Female	an unidentified breed or hybrid	B	G
25	16506-032	Male	Japanese domestic Cats	B	G
26	16640-033	Male	Scottish Fold	B	G
27	17379-034	Female	an unidentified breed or hybrid	B	G
28	17487-035	Female	an unidentified breed or hybrid	B	G
29	783-036	Female	Scottish Fold	B	G
30	17619-037	Female	Scottish Fold	B	G
31	11019-039	Male	American Shorthair	B	G
32	14743-040	Male	Maine Coon	B	G
33	GA-042	Male	Japanese domestic Cats	B	G
34	18852-043		an unidentified breed or hybrid	B	G
35	18410-044	Male	an unidentified breed or hybrid	B	G
A family of Ragdll cat with blood type AB					
36	AHB-26	Male	Ragdoll (Proband)	AB	G
37	AHB-26-32	Female	Ragdoll (Mother)	AB	G
38	AHB-26-33	Male	Ragdoll (Father)	A	G

F father M: Mother, G:Genomic organization, C:cDNA analysis
