# Supplementary material for: Molecular Characterization of the Cytidine Monophosphate-N-Acetylneuraminic Acid Hydroxylase (CMAH) Gene Associated with the Feline AB Blood Group System
Source: PLoS One. 2016 Oct 18;11(10):e0165000. doi: 10.1371/journal.pone.0165000 (PMC5068781; doi:10.1371/journal.pone.0165000)
Supplement: S2 Table — (PDF) [file pone.0165000.s002.pdf]

S2 Table. Primer sequences and PCR conditions.

Region		Primer sequences (5'-3')	Location	Genbank Ac No or Ref No	Initial denature (℃/min)	Denature (℃/Sec)	Annealing (℃/Sec)	Extension (℃/Sec)	Cycle	Final extension (℃/min)
**cat CMAH cDNA**										
E1a/E5	F	GAGCTGTTTTGTGCTGTTTGAC	5' UTR in E1a	EF127684	95/4	95/60	55/60	72/60	35	72/7
	R	CTCAGGTGGTCTGAGTGCATG	E5	EF127684						
E3/E11	F	TCAAGACGAACTAGTTGTTGA	E3/E4	EF127684	95/4	95/60	55/60	72/60	35	72/7
	R	CCTGACCACCAGGTTGTAATCC	E11	EF127684						
E9/E15	F	GAAGCTCTTGAACTACAAGGC	E9	EF127684	95/4	95/60	55/60	72/60	35	72/7
	R	AATTTTCTATGTTGTTTGGCATCT	3'UTR (E15)	EF127684						
E1b /E2	F	AACTCACGAAGTAGCTAGCT	5' UTR in E1b	[13]	95/4	95/60	55/60	72/60	35	72/7
	R	TCCTTGATGCTTGCACACGT	E2	EF127684						
**cat CMAH genomic DNA**										
E1a /Int1a	F	TGAAATTTACAGGCTGGAGT	5' UTR in E1a	NC_018727.2	95/4	95/60	58/30	72/30	35	72/7
	R	TAGCAAACATATCAATCCCA	Int1a	NC_018727.2						
E1b/Int1b	F	AGATACACCGCGTGACAGG	5' UTR in E1b	NC_018727.2	95/4	95/60	60/15	72/60	35	72/7
	R	CCTGGAAATGCACGCTCTGC	E1b/Int1b	NC_018727.2						
Int1b/E2/Int2	F	GCTCTCCTAAGTCTTAACG	Int1b	NC_018727.2	95/4	95/60	62/30	72/30	35	72/7
	R	AATTTCCCAGTGAGCGCAC	Int2	NC_018727.2						
Int2/E3/Int3	F	GCCAAGTAATGCACTCAAGC	Int2	NC_018727.2	95/4	95/60	62/30	72/30	35	72/7
	R	GAGTAGAGACACAAGCTGAC	Int3	NC_018727.2						
Int3/E4/Int4	F	TTGGCGTTGTCTGTCTCCAG	Int3	NC_018727.2	95/4	95/60	62/30	72/30	35	72/7
	R	ATAGCTCCCTCAGAATATTCG	Int4	NC_018727.2						
Int4/E5/Int5	F	TCTATCCTAACGAAGCAGCG	Int4	NC_018727.2	95/4	95/60	62/30	72/30	35	72/7
	R	TGACCTGAGTCCGCTTCTTC	Int5	NC_018727.2						
Int5/E6/Int6/E7/Int7	F	TGACCTGAGCTGAAGTCAGG	Int5	NC_018727.2	95/4	95/60	62/30	72/30	35	72/7

	R	CACTGTTCTAAGGCAAGGAC	Int7	NC_018727.2						
Int7/E8/Int8	F	AAACAGACATACATGGGAGAC	Int7	NC_018727.2	95/4	95/60	58/30	72/30	35	72/7
	R	CTGGTTTCCCAGTTAAGACTG	Int8	NC_018727.2						
Int8/E9/Int9	F	TTTTCACTCATGACACAGAGC	Int8	NC_018727.2	95/4	95/60	58/30	72/30	35	72/7
	R	GGATTCTAGCTGCCAAGCAC	Int9	NC_018727.2						
Int9/E10/Int10	F	AATTAGGGACATTAGGCCAAG	Int9	NC_018727.2	95/4	95/60	58/30	72/30	35	72/7
	R	TGATTCCATGAGAATCTCAGC	Int10	NC_018727.2						
Int10/E11/Int11	F	CGTTAACGTCCCGCATAACC	Int10	NC_018727.2	95/4	95/60	58/30	72/30	35	72/7
	R	GTGGACCTGAGGGAACATGC	Int11	NC_018727.2						
Int11/E12/Int12	F	GGAGGGACTATACGAACTC	Int11	NC_018727.2	95/4	95/60	58/30	72/30	35	72/7
	R	AGTTGCGACAACTAGCACAG	Int12	NC_018727.2						
Int12/E13/Int13	F	TTCTCCCAGGGACATGAGGG	Int12	NC_018727.2	95/4	95/60	62/30	72/30	35	72/7
	R	ACAGTCAGTCCCTGACTCTG	Int13	NC_018727.2						
Int13/E14/Int14	F	GGTCCGCATTCAACACACTG	Int13	NC_018727.2	95/4	95/60	58/30	72/30	35	72/7
	R	AATGTTAGCTCTTCTCACTCC	Int14	NC_018727.2						
Int14/E15/Int15	F	CGTATAGTTCAGTTCACAACC	Int14	NC_018727.2	95/4	95/60	58/30	72/30	35	72/7
	R	CCTGACTCTCTTAATGGACC	3'UTR (E15)	NC_018727.2						
**Additional primers**										
sequence primer	F	CCAAATGTTCAGGAGATCTGG	E4	EF127684						
sequence primer	R	AGAGCGGTGTCCAGTTGACCA	E6	EF127684						
sequence primer	F	AGCCGGGCCTTGTAGTTCAAG	E9	EF127684						
sequence primer	R	ATTTAAGGATTACAACCTGGT	E11	EF127684						
sequence primer		TGAAGTATTCTTTTATCCAGG	E11	EF127684						
M13	F	GTAAACGACGGCCAG	pCR™4-TOPO							
	R	CAGGAAACAGCTATGAC								

F:forward primers, R:reverse primers
