# Supplementary material for: Molecular Characterization of the Cytidine Monophosphate-N-Acetylneuraminic Acid Hydroxylase (CMAH) Gene Associated with the Feline AB Blood Group System
Source: PLoS One. 2016 Oct 18;11(10):e0165000. doi: 10.1371/journal.pone.0165000 (PMC5068781; doi:10.1371/journal.pone.0165000)
Supplement: S3 Table — (PDF) [file pone.0165000.s003.pdf]

S3 Table. Distribution of feline AB blood group antigens among the randomly selected cats.

Animals	N	Type A Neu5Gc	Freq. %	Type B Neu5Ac	Freq. %	Type AB Neu5Gc Neu5Ac	Freq. %
**Pure bred**							
Japanese domestic Cats	86	78	90.7	8	9.3	0	0.0
American Shorthair	59	57	96.6	2	3.4	0	0.0
Scottish Fold	32	27	84.4	5	15.6	0	0.0
Maine Coon	24	22	91.7	2	8.3	0	0.0
Persian	17	15	88.2	2	11.8	0	0.0
Abyssinian	13	12	92.3	1	7.7	0	0.0
Russian Blue	13	13	100.0	0	0.0	0	0.0
Norwegian Forest Cat	7	7	*	0	*	0	*
American curl	5	5	*	0	*	0	*
British Shorthair	5	5	*	0	*	0	*
Somali	5	5	*	0	*	0	*
Ragdoll	4	4	*	0	*	0	*
Singapura	4	4	*	0	*	0	*
Birman	3	3	*	0	*	0	*
Ocicat	2	2	*	0	*	0	*
Himalayan	2	2	*	0	*	0	*
Exotic Shorthair	1	1	*	0	*	0	*
Egyptian Mau	1	1	*	0	*	0	*
Oriental Shorthair	1	1	*	0	*	0	*
Siberian	1	1	*	0	*	0	*
Siamese	1	1	*	0	*	0	*
Chartreux	1	1	*	0	*	0	*
Tonkinese	1	0	*	1	*	0	*
Burmese	1	1	*	0	*	0	*
Bengal	1	1	*	0	*	0	*
Munchkin	1	1	*	0	*	0	*
Total of Pure-bred cats	291	270	92.8	21	7.2	0	0.0
Non pure-bred **	482	465	96.5	17	3.5	0	0.0
Total of animals	773	735	95.1	38	4.9	0	0.0

*frequency do not shows in breed less than 10 animals.

** unidentified breed or hybrid
