# Supplementary material for: Molecular Characterization of the Cytidine Monophosphate-N-Acetylneuraminic Acid Hydroxylase (CMAH) Gene Associated with the Feline AB Blood Group System
Source: PLoS One. 2016 Oct 18;11(10):e0165000. doi: 10.1371/journal.pone.0165000 (PMC5068781; doi:10.1371/journal.pone.0165000)
Supplement: S4 Table — (PDF) [file pone.0165000.s004.pdf]

S4 Table. Frequency of diplotypes and individuals in cats with blood type B.

Diplotypes	Haplotypes	N	Frequency	Breed
1	1-1	0	0.000	
2	2-2	19	0.559	Japanese domestic Cats(4), Scottish Fold(2),Maine Coon(1), Persian(1), Tonkinese(1), an unidentified breed or hybrid (10)
3	2-5	5	0.147	Scottish Fold(1), an unidentified breed or hybrid (4)
4	2-3	4	0.118	Abyssinian(1),Japanese domestic Cats(1),Scottish Fold(1), an unidentified breed or hybrid (1)
5,6	2-4	2	0.059	Japanese domestic Cats(1), American Shorthair(1)
7	3-8	1	0.029	Japanese domestic Cats(1)
8	4-7	1	0.029	American Shorthair(1)
9	3-7	1	0.029	an unidentified breed or hybrid (1)
10	3-9	1	0.029	an unidentified breed or hybrid (1)
Total		34	1.000	

( ) : number of animals
