# Supplementary material for: Molecular Characterization of the Cytidine Monophosphate-N-Acetylneuraminic Acid Hydroxylase (CMAH) Gene Associated with the Feline AB Blood Group System
Source: PLoS One. 2016 Oct 18;11(10):e0165000. doi: 10.1371/journal.pone.0165000 (PMC5068781; doi:10.1371/journal.pone.0165000)
Supplement: S5 Table — (PDF) [file pone.0165000.s005.pdf]

S5 Table. Summary of diplotypes and haplotypes in a family of ragdoll cats with blood group AB.

Diplotypes	Haplotypes	Individual	Breed	Blood type
11	4-6	Male proband	Ragdoll	AB
12	2-6	Mother	Ragdoll	AB
13	1-4	Father	Ragdoll	A
